# P–N Heterojunction System Eu‐Doped ZnO@GO for Photocatalytic Water Splitting

**DOI:** 10.1002/gch2.202200106

**Published:** 2023-01-22

**Authors:** Neeta Gurbani, Neelu Chouhan

**Affiliations:** ^1^ Department of Pure and Applied Chemistry University of Kota MBS Road Kota 324005 India

**Keywords:** europium‐doping, graphene oxide, hydrogen generation, water splitting, ZnO

## Abstract

Here, a feather‐like Eu‐doped ZnO (particle size ≈ 34.87 µm and *E*_g_ ≈ 3.13 eV) nanoassembly is synthesized by using the capping agent cetyltrimethylammonium bromide‐supported hydrothermal method. The Eu‐doped ZnO is loaded onto the graphene oxide (GO) surface as Eu‐doped ZnO@GO (particle size ≈ 23.07 µm and *E*_g_ ≈ 0.79 eV) and applied to measure the photocatalytic water splitting activity in 20% CH_3_OH under a 300 W Xe light source. Eu‐doped ZnO@GO exhibits the higher hydrogen generation activity of 255.8 µmol h^−1^ g^−1^ that is 159 and 1.5 times more than the pristine GO and Eu‐doped ZnO systems, respectively. Eu‐doped ZnO enhances the photocatalytic activity of GO because the p–n junction formed between GO and Eu‐doped ZnO might support the charge‐transfer and suppress charge recombination. The light harvesting power of Eu‐doped ZnO@GO makes the charge transfer smooth through the GO network. Surface photovoltage and electrochemical impedance studies of the Eu‐doped ZnO@GO composite, reveal that GO acts as the p‐type semiconductor and Eu‐doped ZnO works as an n‐type semiconductor and their interface facilitates the p–n junction to ease charge separation and results in enhanced the water‐splitting efficiency.

## Graphical abstract

Eu‐doped ZnO@GO (graphene oxide) with p–n junction is fabricated by a hydrothermal method and used for photocatalytic water splitting. The Eu‐doped ZnO@GO composite exhibits 159 and 1.5 times higher rate of hydrogen generation than the pristine GO and Eu‐doped ZnO systems, respectively. Here, GO acts as the p‐type and Eu‐doped ZnO worked as an n‐type semiconductor to suppress the charge‐carrier's recombination.

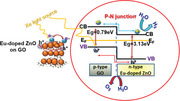

## 1 Introduction

Graphene has a 2D‐honeycomb nanostructure, which attracted the intensive attention of researchers due to its outstanding properties such as ultrathin structure, hardness (strength 42 N m^−1^), toughness (Young's Modulus 1.00 T Pa^−1^), optical transparency, high current conductivity (1 × 10^8^ Ω^−1^ m^−1^), high carrier mobility (200 000 cm^2^ V^−1^ s^−1^) at room temperature and large surface area (2630 m^2^ g^−1^).^[^ ^1^ ^]^ In addition to the above, graphene can uniformly absorb light over a broad spectrum of wavelengths, from infrared to ultra violet via the visible region. The chemical functionalization of graphene also provides a different method for adjusting its chemical characteristics.^[^ ^2^, ^3^ ^]^ Graphene oxide (GO) is the most prevalent derivative of graphene, created by vigorously oxidizing graphite^[^ ^4^, ^5^, ^6^ ^]^ and is thought of as a single sheet of graphene that is retaining different oxygen‐containing groups on both sides of the basal plane as well as on the edges.^[^ ^7^ ^]^ Due to the creation of sp^3^ hybridized C‐atoms, which interfere with the π‐conjugation in the graphene sheet, GO functions differently from pure graphene and acting as an insulator or shows the p‐type semiconducting behavior.^[^ ^8^, ^9^, ^10^ ^]^ It has been shown that the conduction band (CB) minimum of GO is composed of π* orbitals, which have a higher energy level than that needed for hydrogen generation. Graphene and graphene‐based materials are currently being used vigorously for energy conversion applications, including the solar hydrogen generation from water splitting.^[^ ^11^, ^12^ ^]^ There have been numerous attempts to improve the photocatalytic performance of semiconductor photocatalysts by integrating graphene with them for various applications.^[^ ^13^, ^14^, ^15^, ^16^, ^17^, ^18^ ^]^ ZnO is an inexpensive, wide‐bandgap semiconductor with a bandgap of 3.37 eV, which corresponds to a wavelength of about 375 nm. Its high photoreactivity, good photostability, and low cost favor its use in photocatalytic water splitting (PWS), but its limitations include photocorrosion, backward reaction, poor stability, and inability to absorb visible light.^[^ ^19^ ^]^ To get around these problems, modifications have been made like coupling with other semiconductors and doping with metal, rare‐earth metal, or non‐metal ions.^[^ ^20^, ^21^, ^22^, ^23^ ^]^ Recently, rare‐earth metal‐doped ZnO has caught the attention of material scientists in this direction because of its superior activity and selectivity to pure ZnO as well as its higher absorption efficiency in the visible region, which can be used to create better optoelectronic devices for harvesting visible light. The dopant causes competitive defects in the ZnO surface, bulk, and lattice. Due to their role in the bandgap contraction and the separation effect of the photocarriers (photoelectron (e^−^) and photohole (h^+^)), these defects are wanted at the surface.

The imperfectly filled 4f orbitals of rare‐earth metals can trap electrons, reducing charge recombination and enhancing photocatalytic activity.^[^ ^23^ ^]^ By interacting with 4f orbitals of lanthanoid ions with a variety of functional groups of GO, the light harvesting power of composite will enhanced. In comparison to pure TiO_2_, Khalid et al. studied the La/TiO_2_‐graphene composites revealed higher visible light absorption and improved charge separation capability. Ahmad et al.^[^ ^25^ ^]^ synthesized europium and terbium ‐doped ZnO photocatalyst and found the extended spectral response from UV to visible range and reducing the bandgap from 3.25 to 2.91 eV. The photocatalytic hydrogen evolution activity over Eu and Tb codoped ZnO was significantly increased to 533.8 and 792 µmol with 0.2 wt% catalysts and pH = 9. In the study of the Eu^3+^, Pr^3+^, and Yb^3+^‐doped TiO_2_, Ranjit et al.^[^ ^26^ ^]^ noted an increase in the TiO_2_ photocatalyst's capacity in solutions containing salicylic acid and cinnamic acid. A few of the Eu‐doped systems for water splitting have been mentioned in **Table** **1**; however, their use for hydrogen generation for water splitting is relatively limited. Eu‐doped nanocomposites have been researched for dye degradation and the removal of various metal ions.

**Table 1:** Summary of photocatalytic hydrogen generation activity of lanthanum‐based nanocomposites

S. no.	Photocatalyst	Light source	Amount of H_2_ generation [µmol g^−1^ h^−1^]	Amount of O_2_ generation [µmol g^−1^ h^−1^]	Reference
1.	Eu‐doped TiO_2_	Visible light	2460	1236	[27], 2008
2.	Eu‐TiO_2_/GO	λ > 400 nm	100 000	–	[24], 2012
3.	Eu‐Tb‐doped ZnO	λ > 400 nm	533.8/792.0 (pH = 9)	–	[25], 2020
4.	Tb‐Sm codoped ZnO/CNT	Visible light	2683	–	[28], 2022
5.	rGO‐Er‐TiO_2_	Visible light	115.37	–	[29], 2021
6.	Ag_3_PO_4_/TiO_2_/GO	**–**	218.70	–	[30], 2018
7.	Ag_3_PO_4_@GO	300 W Xe lamp	**–**	1620	[31], 2017
8.	Ag_3_PO_4_@AgCl/GO	300 W Xe lamp	–	34.5	[32], 2015
9.	This work (Eu‐doped ZnO@GO)	300 W Xe lamp	229.0	–	2022

In this paper, we synthesized the Eu‐doped ZnO‐loaded GO and applied them for photocatalytic water splitting. Here, we used the hydrothermal method for synthesis of Eu‐doped ZnO assembly and Hummer's method to synthesize GO.

## 2 Characterization

The GO, Eu‐doped ZnO nanoassembly and Eu‐doped ZnO@GO nanocomposite were analyzed for the powder X‐ray diffraction (XRD) by using the diffractometer (Rikagu Ultima‐IV type II model; the copper target (Cu*K*_α_ radiation) and ultrafast detector in reflection mode) established at the University of Kota, Kota (India) in the range of 2θ = 05°–60/80 °C at 45 mA current and 30 mV voltage. The morphology and elemental composition of the compounds were analyzed by using Field Emission Scanning electron microscopy (FESEM) microscopy (SEM; JEOL, JEM 2100) and FESEM‐supported Energy Dispersive X‐ray spectroscopy (EDX) (Oxford instrumentation) at MNIT, Jaipur (India). UV–vis spectrophotometer (Perkin Elmer, Lambda 750, USA bench‐top Model) with double‐beam and the double monochromatic facility was used for investigation at Malaviya National Institute of Technology (MNIT), Jaipur (India). The steady‐state photoluminescence emission (PLE) spectra were documented at excitation energy of wavelength 380 nm by using a Perkin Elmer (LS‐55) Fluorescence Spectrophotometer at MNIT, Jaipur (India). Ex situ X‐ray photoelectron spectroscopy was performed at MNIT, Jaipur (India) by using a spectrophotometer (Omicron Multiprobe Surface Analysis System, Germany, Gmbh at ultrahigh vacuum 5 × 10^−11^ Torr, at Zn 3d (1022.0 eV), Eu 3d (1140.0 eV) and Eu 4d (140.0 eV), C 1s (284.6 eV) and O 1s (532.0 eV), respectively, at MNIT, Jaipur (India). Surface photovoltaic spectroscopic (SPS) measurements were conducted at Nanoscale Research Facility (NRF) IIT, New Delhi using the Surface photovoltaic (SPV) instrument (make: Kelvin Probe (KP) Technology, Scotland; model: APS04‐N2‐RH) at room temperature (RT) under visible light exposure using a vibrating gold Kelvin probe (Delta PHI Besocke) mounted inside a home‐built vacuum chamber.

The SPV samples were prepared by drop‐casting of the aqueous dispersion of 0.5 mg catalyst onto 1 × 1 cm^2^ glass substrates that was dried in air and overnight annealed at 70 °C. Samples were illuminated with monochromatic light from a 175 W Xe lamp filtered through an Oriel Cornerstone 130 monochromator and the light intensity range is 0.1 to 0.3 mW cm^2^.

The electrochemical impedance spectroscopy (EIS) study of the studied systems were performed by using the electrochemical working station (Potentiostat/Galvanostat Autolab‐204 Metrohm, Netherlands) using a conventional three electrodes cell. Where, GO, Eu‐doped ZnO, Eu‐doped ZnO @GO casted electrodes were used as working electrodes, graphite rod as counter electrode, and Ag/AgCl electrode in 3 m KCl were used as reference electrode dipped in an aqueous 20% CH_3_OH. The EIS measurements were carried out in the frequency range of 100 kHz to 0.1 Hz by applying an AC voltage with ±10 mV perturbation.

### 2.1 Photocatalytic Hydrogen Production

The powder photocatalyst (0.30 g) was dispersed in 120 mL of aqueous methanol (20% CH_3_OH of pH = 7.0) kept in a reaction cell (volume ≈150 mL, Pyrex glass with water jacket for maintaining the reaction temperature 25 °C). The reaction vessel was tightly air‐sealed with the help of a rubber septum and plastic wire lock. The suspension of the compound was purged with Ar‐gas for 1 h by adjusting the reaction cell pressure 1 atmosphere to expel the air from the reaction vessel. Then, the suspension was irradiated with the 300 W Xe lamp (Xe lamp‐HX1, ISS‐ Model PE300UV; >420 nm, light intensity 1 × 10^21^ photons per hour). As‐resulted gaseous products were half‐hourly monitored for 3.5 h through an inverted gas collection graduated cylinder by displacement of water from a water‐fill. The gaseous products were analyzed with the help of the gas chromatograph (Shimadzu, Thermal conductivity Detector (TCD) along with 5A columns molecular sieve).

## 3 Result and Discussions

### 3.1 Synthesis and Morphology

The hydrothermal method was used to prepare the nanohybrid Eu‐doped ZnO by taking 10% of europium doping to ZnO in 20% cetyltrimethyl ammonium bromide (CTAB) surfactant. Modified Hummer's method was used to fabricate the graphene oxide. The improvised approach of the synthesis of the Eu‐doped ZnO@GO was used, which might follow the below mentioned growth route.^[^ ^33^ ^]^

(1) $$\begin{array}{c} Zn^{2+}+4{\text{OH}}^{-}\to {\left[Zn{\left(OH\right)}_{4}\right]}^{2-} \end{array}$$

(2) $$\begin{array}{c} {\left[Zn{\left(OH\right)}_{4}\right]}^{2-}\to Zn{\left(OH\right)}_{2}+2{\text{OH}}^{-} \end{array}$$

(3) $$\begin{array}{c} Zn{\left(OH\right)}_{2}\to ZnO+H_{2}O \end{array}$$

(4) $$\begin{array}{c} \begin{array}{l} {\left(CTA\right)}_{2}^{+}-{\left[Zn{\left(OH\right)}_{4}\right]}^{2-}+2{\text{Br}}^{-}+{\text{Eu}}^{3+}\to \\ \;2CTAB+{\text{Eu}}^{3+}-doped\;ZnO+2H_{2}O+2{\text{OH}}^{-} \end{array} \end{array}$$

(5) $$\begin{array}{c} \begin{array}{l} CTAB\;capped\;Eu^{3+}-doped\;ZnO+GO\to \\ \;Eu^{3+}-doped\;ZnO@GO \end{array} \end{array}$$

**Figure 1:**
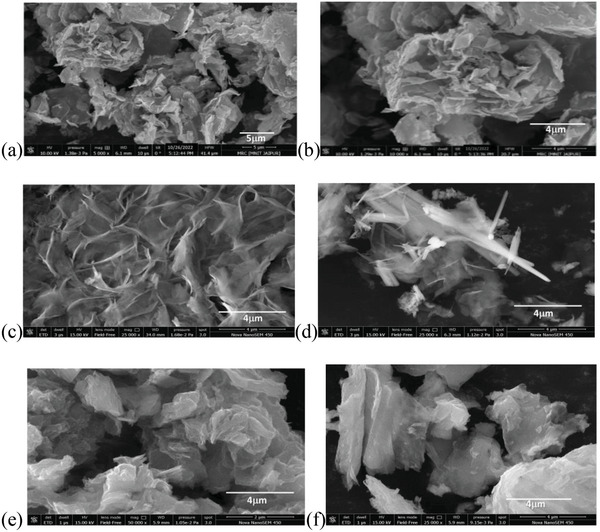
FESEM images for a,b) GO, c,d) Eu‐doped ZnO, and e,f) Eu‐doped ZnO@GO.

Here, the elemental zinc existed as negatively charged tetrahyroxide ion [Zn(OH)_4_]^2−^ that were formed as the product of reaction between Zn^2+^ and OH^−^, where CTA^+^ had a positively charged head with a hydrophobic tail. It was found that in the CTAB‐assisted hydrothermal process, (CTA^+^)_2_‐[Zn(OH)_4_]^2−^ ion pairs were formed initially due to the electrostatic interaction of CTA^+^ and[Zn(OH)_4_]^2−^ ions. The presence of CTAB also accelerates the ionization of [Zn(OH)_4_]^2−^ as strong‐acid‐weak‐base salt (Zn(OH)_2_) that dissociated into ZnO and H_2_O. The (CTA^+^)_2_‐[Zn(OH)_4_]^2−^ ion pairs with Eu^3+^ formed a CTAB capped Eu^3+^‐doped ZnO. It was concluded that the CTAB micelles aggregated to envelope the Eu‐doped ZnO crystallites during the hydrothermal crystallization process and on washing the material with ethanol, the uniform feather‐like structures of CTAB capped Eu^3+^‐doped ZnO were prepared. As‐prepared CTAB capped Eu^3+^‐doped ZnO (Eu‐doped ZnO) tethered with GO surface through the free OH present on the surface of Eu‐doped ZnO. The curved layered (≈0.26 µm thickness) accumulated as flower like structures (particle size ≈15 µm) of GO was observed, as shown in **Figure** **1**a,b. The feather‐like morphology (Figure 1c,d) of the regular artifacts of size particle size ≈34.87 µm and thickness of 0.138 µm was detected for the nanohybrid Eu‐doped ZnO. These layered artifacts aggregated into a few 1D structures as shown by Figure 1d. As‐synthesized nanohybrid Eu‐doped ZnO assembles were loaded on the GO sheets by impregnation method to form nanocomposite, i.e., Eu‐doped ZnO@GO that exhibited the folded layers (particle size ≈23.07 µm thickness ≈0.095 µm) as illustrated by Figure 1e,f. It can be concluded that the thickness of layer decreases with advancement of GO (**Table** **2**).

**Table 2:** FESEM supported EDX analysis of GO, Eu‐doped ZnO and Eu‐doped ZnO @ GO nanocomposites

Sample	Zn%	O%	C%	Eu%
GO	–	03.65	94.98	–
Eu‐doped ZnO	34.58	29.16	35.08	01.10
Eu‐doped ZnO @ GO	02.77	16.66	79.35	01.21

**Figure 2:**
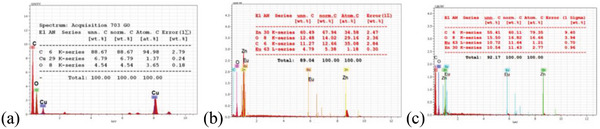
Elemental mapping of a) GO, b) Eu‐doped ZnO nanoassembly, and c) Eu‐doped ZnO @GO nanocomposite. GO sample loaded on Cu grid, thus Cu appeared in EDX of GO.

The FESEM‐supported EDX profile of the GO, Eu‐doped ZnO and Eu‐doped ZnO@GO was observed and mentioned in **Figure** **2**a–c and Table 2. The elemental composition of above systems are demonstrated by along the respective binding energy of Zn(*K*_α_ = 8.63 KeV, *L*_α_ = 1.012 KeV), C(*K*_α_ = 0.277 KeV), O(*K*_α_ = 0.5 KeV), and Eu (*L*_α_ = 5.86 KeV, *M* = 1.31 KeV). The above study confirms the formation of nanocomposite of Eu‐doped ZnO with GO.

### 3.2 XRD Studies

**Figure 3:**
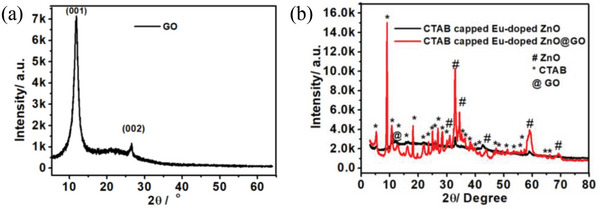
X‐ray diffraction (XRD) patterns of a) GO and b) Eu‐doped ZnO and Eu‐doped ZnO@GO. Addition of surfactant shows significant changes in the XRD pattern.

The XRD patterns of GO, Eu‐doped ZnO, and Eu‐doped ZnO@GO are shown in **Figure** **3**a,b. The major XRD peak of GO was observed at 2θ angles 11.13° (100), which confirm the formation of GO from graphite and the minor peak detected at 26.70° (002) due to the reduction of the GO (Figure 3a). The periodical pattern of Eu‐doped ZnO was found with the presence of CTAB. Figure 3b represents the typical XRD patterns of the composite Eu‐doped ZnO(red) at 5.20°, 10.84°, 11.22°, 14.74°, 16.18°, 22.06°, 24.28°, 33.06°, 34.82°, 42.64°, 58.92°, and 68.44° angles. The reflections observed at the angle 2θ = 31.10°, 33.06°, 34.46°, 43.98°, and 59.16°, correspond to (100), (002), (101), (102), and (110) planes of the hexagonal wurtzite phase of ZnO (matches well with the standard Joint Committee on Powder Diffraction Standards (JCPDS) Card No. 79‐2205) with space group *p63mc*. The rest of the peak belongs to the CTAB molecules that remain unaffected even after the hydrothermal reaction. But CTAB peaks are significantly suppressed on loading of GO and without any additional new peaks, which confirms the existence of the CTAB molecule exist in the same crystal phase. The incorporation of Eu‐doped ZnO into in GO the composite was confirmed by a blueshift in the XRD pattern with respect to the pure counterparts, i.e., Eu‐doped ZnO and GO Figure 3b (black).

The samples’ reported crisp and narrow diffraction peaks supported the compound's good crystallinity.^[^ ^34^, ^35^, ^36^, ^37^ ^]^ Using the Scherrer formula, the corresponding thickness of the graphitic layer was determined for the GO, Eu‐doped ZnO, and Eu‐doped ZnO@GO as 5.88, 34.87, and 23.07 µm, respectively. Which was compatible with the particle size discovered from the SEM investigations.

### 3.3 Optical Study: UV–vis Spectroscopy

**Figure 4:**
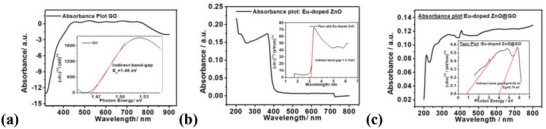
UV–vis absorption analysis of a) GO, b) Eu‐doped ZnO, and c) Eu‐doped ZnO@GO composite along with Tauc plots along with the indirect bandgap.

**Figure** **4**a,b shows the UV–vis absorption spectra of the GO, Eu‐doped ZnO, and composite Eu‐doped ZnO@GO, which were studied in the wavelength range of 200–800 nm and used to determine the bandgap and electronic transitions. The GO spectrum has peaks at 369.48, 425.75, 526.30, 607.60, and 681.16 nm wavelength. The indirect bandgaps of the nanoassembly GO, Eu‐doped ZnO, and nanocomposite Eu‐doped ZnO@GO composite were found in the Tauc plots of the aforementioned systems and are calculated to be 1.46, 3.13, and 0.79 eV, respectively. Additionally, it was demonstrated that the bandgap of the nanocomposite is decreased by the addition of GO to the Eu‐doped ZnO nanoassembly.

The optical absorption of the compound observed at the wavelength 206.07 nm might be attributed to the inter‐band transition of the deeper level valance electrons to the uppermost shell (conduction band).^[^ ^38^ ^]^ This conduct of the Eu‐doped ZnO was appeared due to internal electric fields within the crystal and inelastic scattering of charge carriers by phonons.^[^ ^39^, ^40^ ^]^ An excitonic peak found around 289.98 nm due to the ZnO nanoparticles, which lie much below the band gap wavelength of 334.84 nm (*Eg* = 3.70 eV). It is also evident that significant sharp absorption of ZnO indicates the dispersed hetero‐nature of the nanoparticle distribution.^[^ ^41^ ^]^ The band at 375.24 nm (3.30 eV) corresponds mainly to transitions between the valence band–conduction band of the ZnO, and related to the band gap. Where, the deconvoluted peaks at 396.61 nm and 431.83 nm observed due to the d‐d transition and peaks at 692.44 and 732.00 nm due to the f‐f transition of Eu.

**Figure 5:**
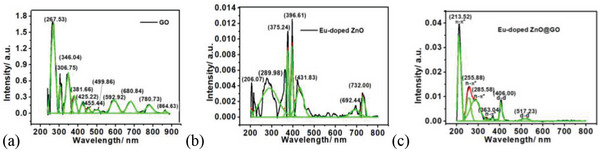
Deconvoluted UV–vis spectra of a) GO, b) Eu‐doped ZnO, and c) Eu‐doped ZnO@GO composite.

The Eu‐doped ZnO@GO composite exhibited the deconvoluted peaks at 213.52, 255.88, 285.58, 363.04, 406.00, and 517.23 nm. Which inferred that the optical absorption of the compound determined at wavelength 230 nm dominated by the π–π* transition.^[^ ^42^ ^]^ These π–π* plasmon peak transition was consequences of the two conjugative effect: one devoted to nanometer‐scale sp2 clusters, and the other due to the chromophore units (C=C, C—O and C=O bonds.^[^ ^43^, ^44^, ^45^, ^46^ ^]^ If the characteristic π–π* plasmonic feature appeared at 213.52 nm due to the sp2 clusters of a single phenyl ring, then the spectral changes in GO, are subject to the effect of the chromophore aggregation.^[^ ^47^, ^48^, ^49^, ^50^, ^51^ ^]^ The excitonic peaks around 255.88 nm and 363.04 eV developed due to the ZnO nanoparticles, Here, the deconvoluted peaks centered at binding energy at 285.58 eV attributed to the C=C, carbonaceous bands^[^ ^52^ ^]^ resulted in significant change in optical absorption intensity of Eu‐doped ZnO@GO (**Figure** **5**b). Based on the above facts, it is inferred that the change of the UV‐vis absorption intensity observed for GO is caused by the conjugative effect of chromophore aggregation, which influences the π–π* plasmon peak.

The deconvoluted UV–vis spectral characteristics of the GO, nanoassembly Eu‐doped ZnO, and composite Eu‐doped ZnO@GO are shown in Figure 5a–c, respectively. The Eu‐doped ZnO@GO composite (Figure 5c) exhibited the deconvoluted peaks at 213.52, 255.88, 285.58, 363.04, 406.00, and 517.23 nm.

This conduct of the Eu‐doped ZnO is focused on the inelastic scattering of charge carriers by phonons and internal electric fields within the crystal.^[^ ^39^, ^40^ ^]^ Due to the ZnO nanoparticles, an excitonic peak was discovered at a wavelength of roughly 289.98 nm, which is significantly below the bandgap wavelength of 334.84 nm. It is also obvious that the dispersed heteronature of the nanoparticle distribution is shown by the substantial acute absorption of ZnO.^[^ ^41^ ^]^ The band at 375.24 nm is associated with the bandgap and primarily pertains to transitions between the ZnO's valence band and conduction band. The deconvoluted peaks at 396.61 and 431.83 nm were observed due to the d–d transition. Where, the deconvoluted peaks of the composite at 406.00 nm (d–d), and 517.23 nm (d–d), are responsible for ZnO, Eu^2+^, and Eu^3+^ interactions (Figure 5b). Whereas, the peaks at 692.44 and 732.00 nm were discovered due to the f–f transition of Eu. The redshift in absorption peaks of Eu‐doped ZnO on GO addition confirmed the effective loading of Eu‐doped ZnO@GO nanocomposite.

### 3.4 PL Emission Spectral Study

**Figure 6:**
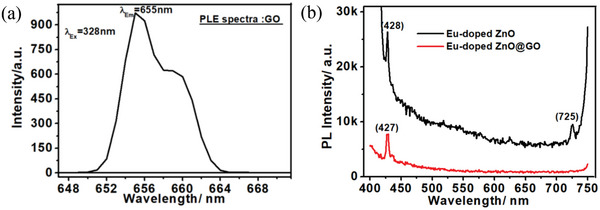
PLE spectra of the a) GO and b) Eu‐doped ZnO and Eu‐doped ZnO@GO.

The photoemission spectra of GO, Eu‐doped ZnO nanoassembly, and Eu‐doped ZnO@GO nanocomposite was registered upon the 380 nm excitations as shown in **Figure** **6**a–c. At 328 nm excitation, the GO displays the red emission with the strong photoluminescence emission (PLE) peaks at 655 nm together with the shoulder peak at 659 nm (Figure 6a). The photoluminescence (PL) spectrum of Eu‐doped ZnO exhibits the characteristic peaks, centered at 428 nm (blue emissions) and 725 nm (red emissions).

**Figure 7:**
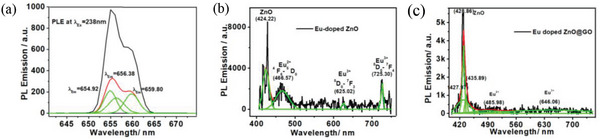
Deconvoluted PLE spectra of the a) GO, b) Eu‐doped ZnO, and c) Eu‐doped ZnO@GO.

The Eu‐doped ZnO powders had a surface flaw that caused the peak at 428 nm to occur (Figure 6b).^[^ ^53^ ^]^ In this PL spectra, the strong peaks at 725 nm are the result of the 4f intratransitions of Eu^3+^ ions (^5^D_0_ → ^7^F_4_), whereas the broad PL band is connected to the flaws in the ZnO lattice.^[^^54]^ These findings imply that during the oxidation process of the side reaction of the conversion of the from Zn(OH)_2_ to ZnO, doped Eu^2+^ ions are transformed into Eu^3+^ ions.^[^ ^55^ ^]^ The Gaussian fitted deconvoluted peaks were observed for GO at 654.92, 656.38, and 659.80 nm (**Figure** **7**a).

The deconvoluted peaks associated with the ZnO, Eu^2+^, and Eu^3+^ ions were found in the Gaussian fitted deconvoluted PLE spectra of the Eu‐doped ZnO nanoassembly shown in Figure 7b at 424.22, 466.57, 625.02, and 725.30 nm, respectively.

Deconvoluted PLE spectra of the GO (Figure 7a) showed peaks associated with the quinone band, pristine band, and vibrational band at 654.92, 658.38, and 659.80 nm, respectively.^[^ ^56^ ^]^

The peaks at 424.22 nm (ZnO), 466.57 nm (^4^F_6_–^5^D_0_ transition of Eu^2+^), 625.02 (^5^D_0_–^7^F_3_ transition of Eu^3+^), and 725.30 nm (^5^D_0_–^7^F_4_ transition of Eu^3+^) were seen in the spectral deconvoluted PL spectra of Eu‐doped ZnO (Figure 7b).

The deconvoluted PL emission spectra of Eu‐doped ZnO@GO nanocomposites, which displayed the broad PLE peak at approximately 427 nm, are shown in Figure 7c. These spectra give deconvoluted peaks at wavelengths of 427.97, 428.86, 435.89, 485.98, and 646.06 nm. Whereas the peaks at 427.97 and 428.86 are associated with ZnO blue emission (UV), the remaining peaks are associated with various kinds of Eu species.

It is well known that the f–d transition caused by the Eu^2+^ ions in crystals often results in a broadband emission at 465 nm.^[^ ^57^ ^]^ The spectra represent transitions from the ground state of the Eu^2+^ ion (^8^S_7/2_) to the lowest excited state (^4^F_6_) ^5^D.^[^ ^58^ ^]^ Through the use of electron spin resonance X‐ray photoelectron spectroscopic (XPS) measurements, we further verified the presence of Eu^2+^ ions in our samples.

The crystal field strength surrounding the Eu^2+^ ions had a significant impact on their capacity for 5d‐excitation.^[^ ^59^, ^60^ ^]^ As shown in Figure 7b,c, the Beevers‐Ross (BR), anti‐BR (a‐BR), and mid‐oxygen (mO) sites of crystal structure are occupied by Eu^2+^ ions. These peaks at 435, 460, and 490 nm correspond to these sites.^[^ ^61^ ^]^ As a result, the Eu‐doped ZnO@GO emission spectra showed deconvoluted Gaussian PL emission peaks at 435.89, 485.98, and 646.06 nm, which were attributed to the Eu^2+^ ions, Eu^2+^ ions, and Eu^3+^ ions, respectively.

### 3.5 XPS Studies

XPS investigations at carbon (1s), oxygen (1s), zinc (2p), and europium (3d and 4p) elements are used to examine the surface chemical environment of the investigated composites and hybrid‐assembly. The C (1s) line of adventitious carbon at 284.6 eV binding energy (BE) was used to normalize the absolute binding energies.^[^ ^62^ ^]^

**Figure 8:**
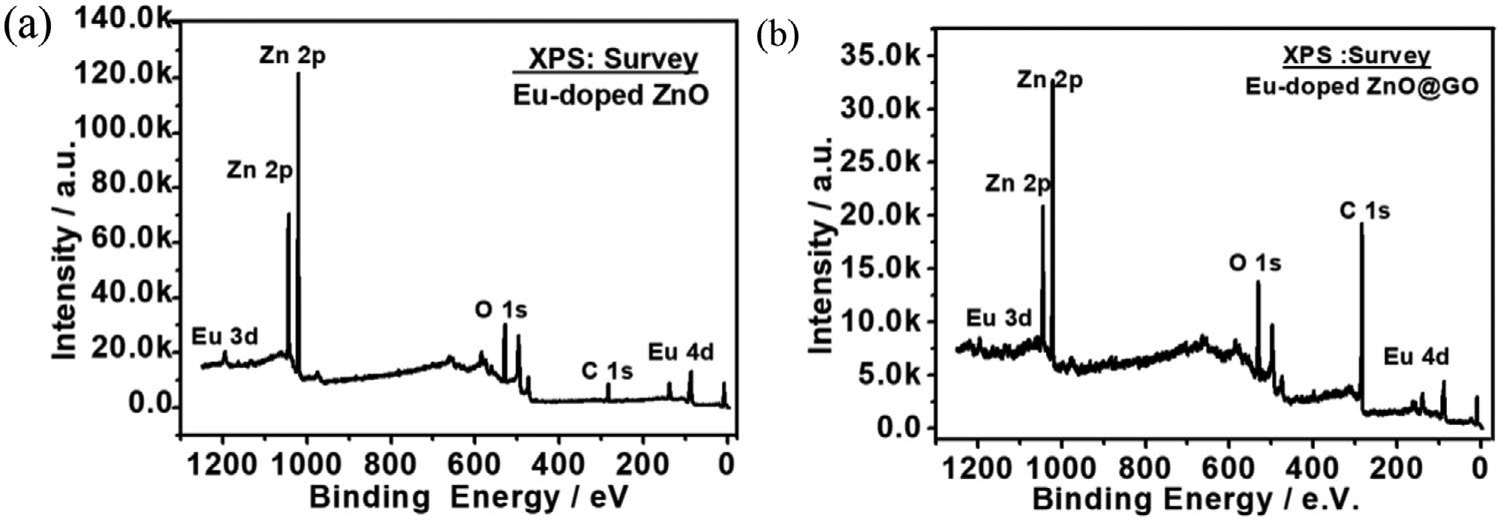
Survey spectra of the a) Eu‐doped ZnO and b) Eu‐doped ZnO@ GO.

When GO, Eu‐doped ZnO, and Eu‐doped ZnO@ GO XPS survey spectra were fairly compared, loading of Eu‐doped ZnO on GO was seen as the increased carbon and oxygen content and decrease Zn and Eu elements in the survey spectra of the Eu‐doped ZnO@ GO, as illustrated in **Figure** **8**a,b.

**Figure 9:**
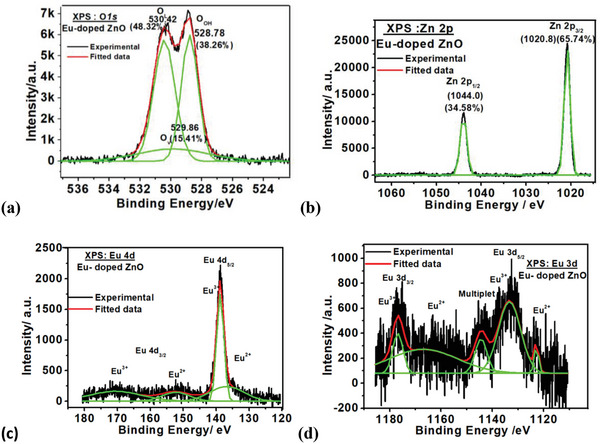
Deconvoluted core level XPS spectra of Eu‐doped ZnO nanocomposite at binding energy of a) O 1s, b) Zn 2p, c) Eu 4d, and d) Eu 3d level.

Figure S1a,b in the Supporting Information exhibits the deconvoluted XPS spectra of GO(unused) at the core levels C 1s, and O 1s, respectively. **Figure** **9**a,d shows the deconvolution XPS binding energy spectra at the core levels O 1s, Zn 2p, and Eu 3d of Eu‐doped ZnO. The observed binding energy spectrum (Figure 9a) of the O 1s core level shows a broad and asymmetric nature, which indicates the presence of more than one form of oxygen in the compound that can be extracted by deconvolution of the O1s peak. Peaks in the deconvoluted O1s spectrum are located at 529.06 eV (17.77%), 529.45 eV (23.45%), and 530.96 eV (58.78%), respectively. These peaks are attributed to the lattice oxygen O_L_, oxygen vacancy Ov, and hydroxyl group oxygen O_OH_.^[^ ^63^ ^]^ The presence of O^2−^ ions in the hexagonal ZnO lattice, which is surrounded by Zn atoms, is responsible for the lowest binding energy peak of O1s at 529.06 eV (17.77%).^[^ ^64^ ^]^ The intermediate peak at 529.45 eV (23.45%) is connected to O^2−^ ions present in oxygen‐deficient regions^[^ ^65^, ^66^ ^]^ of ZnO matrix. The adsorbed OH group on the surface of the nanoassembly of Eu‐doped ZnO typically corresponds to the peak at 530.96 eV (58.78%).^[^ ^67^, ^68^ ^]^

The binding energies at 1044.00 eV (34.58%) and 1020.80 eV (65.74%), which correspond to the Zn 2p_1/2_ and Zn 2p_3/2_ states of the zinc, were found to be the center of the deconvoluted XPS core of symmetric Zn 2p spectra, which is the usual feature of the ZnO systems (Figure 9b). The presence of numerous components of Zn in the samples was ruled out because there was no discernible change in the shape of Zn2p spectra. Furthermore, both samples’ Zn 2p_1/2_ and Zn 2p_3/2_ peak locations nearly matched the conventional values for ZnO.^[^ ^69^ ^]^ Zn was proven to be in the +2 oxidation state by the 23.15 eV^[^ ^70^ ^]^ difference between the Zn 2p_1/2_ and Zn 2p_3/2_ peak positions, which represents the spin split values and the earlier stated valence state of Zn did not significantly alter on Eu addition.^[^ ^69^, ^70^ ^]^

For the Eu‐doped ZnO system, Figure 9c,d shows the deconvulated high‐resolution XPS binding energy spectra at the core levels of Eu 4d and Eu 3d. In continuation with the results obtained for the PLE spectra and absorption spectra, it also confirms the two oxidation forms of Eu (i.e. Eu^2+^ and Eu^3+^). Eu 4d peaks (Figure 9c) were found at binding energy of 136.82 eV (Eu^2+^), 138.78 eV (Eu^3+^), 152.27 eV (Eu^2+^), and 176.65 eV (Eu^3+^). At the binding energies of 1122.9 eV (Eu^2+^), 1133.30 eV(Eu^3+^), 1144.4 eV(multiplet), 1166.9 eV (Eu^2+^), and 1176.8 eV (Eu^3+^), Eu 3d peaks were found. The XPS results supported the PLE findings that Eu‐doped ZnO contained a larger concentration of the Eu^3+^ species than Eu^2+^ species.

**Figure 10:**
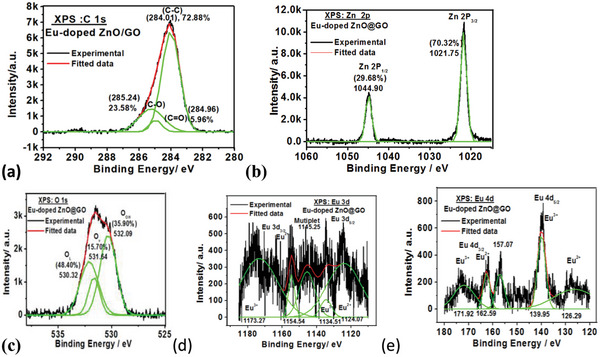
XPS spectra of Eu‐doped ZnO @ GO nanocomposite at BE of a) C 1s, b)Zn 2p, c) O 1s, d) Eu 3d, and e) Eu 4d.

The XPS binding energy spectra of the Eu‐doped ZnO@GO samples are shown in **Figure** **10**a–e at the core levels of C 1s, Zn 2p, O 1s, Eu 3d, and Eu 4d.

Similarly, the Eu‐doped ZnO@GO samples showed the core levels of high‐resolution XPS spectra at C1s binding energy (Figure 10a), with the distinct forms of carbon at 285.24 eV(C—O—C/C—OH; 23.58%), 284.01 eV(C—C/C—H; 72.88%), and 284.96 eV(C—O; 5.96%). It demonstrated that the majority of the carbon in Eu‐doped ZnO@GO belongs to GO. The presence of many forms of oxygen in the molecule was confirmed by the wide and asymmetric O 1s core level binding energy spectrum shown in Figure 9b, which can be recovered by deconvolution of the O1s peak. At BE 530.32 eV (48.40%), 531.64 eV (15.70%), and 532.09 eV (35.90%), the distinct forms of oxygen were seen. These forms were attributed to lattice oxygen (O_L_), vacancy oxygen (Ov), and hydroxy oxygen (O_OH_).^[^ ^61^ ^]^ The redshift in spectra caused by GO addition to Eu‐doped ZnO. Additionally, due to the interaction of Eu‐doped ZnO with the GO matrix, an increase in lattice oxygen was also seen along with a decrease in OH oxygen and an increase in oxygen vacancy.

According to the high‐resolution XPS scans of the symmetrically deconvoluted XPS core levels Zn 2p spectra (Figure 10c), the zinc exists in the Zn 2p_1/2_ and Zn 2p_3/2_ states, respectively, at binding energies of 1044.90 (29.68%) and 1021.75 (70.32%). The Zn 2p_1/2_ and Zn 2p_3/2_ states’ positions differed by 23.20 eV, which is in good agreement with the conventional value of 22.97 eV^[^ ^71^ ^]^ and indicates that zinc present in the 2+ oxidation state. The position of the Zn 2p_1/2_ and Zn 2p_3/2_ states shifted to the higher binding energy on the addition of the GO to Eu‐doped ZnO without any noticeable change in the shape of Zn 2p spectra, which discards the possibility of the multiple valency of Zn. Additionally, the positions of Zn 2p_3/2_ and Zn 2p_1/2_ peaks of Zn in both samples harmonized with the standard ZnO values in close vicinity.^[^ ^69^ ^]^ Moreover, the addition of Eu and GO did not show any remarkable change in the valence state of Zn the same was conveyed by the literature.^[^ ^69^, ^70^ ^]^

Figure 10d,e shows the XPS binding energy spectra at the core levels Eu 4d and Eu 3d, respectively. The core level XPS spectra of Eu‐doped ZnO@ GO exhibited the binding energy of Eu 3d of Eu 3d_5/2_ at 1124.07 eV (Eu^2+^),1134.50 eV (Eu^3+^), 1145.49 nm (multiplets), Eu 3d of Eu 3d_3/2_ at 1154.54 eV (Eu^2+^) and 1173.27 eV(Eu^3+^) and Eu 4d of Eu 4d_5/2_ at 126.30 eV (Eu^2+^) and 139.95 eV (Eu^3+^), 157.07 eV, Eu 4d of Eu 4d_3/2_ at 162.59 eV (Eu^2+^) and 171.92 eV (Eu^3+^).^[^ ^72^ ^]^ The XPS results go hand in hand with the results obtained in the PL study, the higher concentration of the Eu^2+^ species in Eu‐doped ZnO@GO in comparison to the Eu^3+^ species. The XPS study confirms the presence of the Eu^2+^ and Eu^3+^ ions in both studied samples, but both 4d and 3d doublet spectra shifted to the lower energy sites on the addition of the GO to Eu‐doped ZnO. It showed that the presence of GO caused a decrease in Eu^3+^ in Eu^2+^.

**Figure 11:**
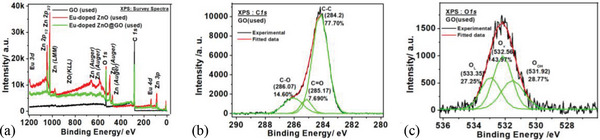
Deconvulated core level XPS spectra of the GO(used) at C1s and O1s.

**Figure 12:**
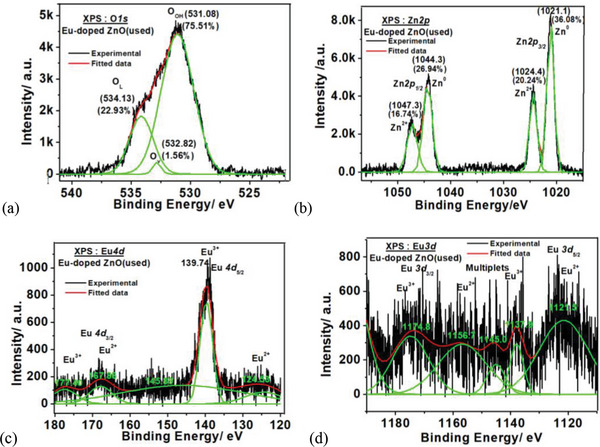
Deconvulated core level XPS spectra of the Eu doped ZnO(used) at BE of O1s, Zn2p, Eu3d, and Eu4d level.

**Figure 13:**
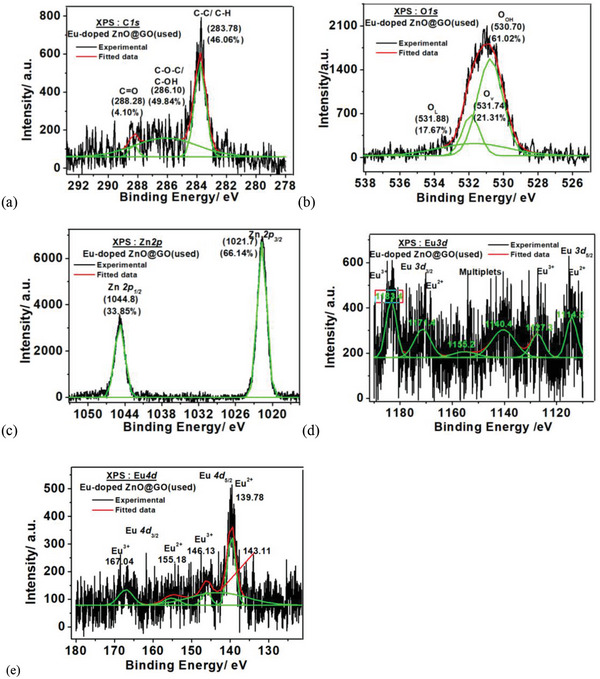
Deconvulated core level XPS spectra of the Eu‐doped ZnO@GO(used) at the BE of C1s, O1s, Zn2p, Eu3d, and Eu4d.

Post water splitting deconvoluted XPS spectral study of GO, Eu‐doped ZnO, and Eu‐doped ZnO@GO was also observed at C1s, O1s, Zn2p, Eu 3d, and Eu 4d core energy levels (**Figures** **11**a–c, **12**a–d, and **13**a–e and **Table** **3**) along with their survey spectra (Figure 11a) and compared with the corresponding unused samples. The redshift was observed for the used sample's XPS spectra with respect to the unused corresponding samples. Interestingly, the double doublet was observed for the Zn2p of the used Eu‐doped ZnO due to the formation of more than one species (i.e. Zn^2+^ and Zn^0^) in spite of the doublet of unused Eu‐doped ZnO (due to the formation of Zn^2+^), where the used Eu‐doped ZnO@GO exhibited only doublet (because of Zn^2+^ formation). That confirms the role of GO in providing extra stability to Eu‐doped ZnO system. The XPS measurements of GO (unused and used) at the binding energies of C1s and O1s was performed. It was noticed that as compared to the unused GO samples, the % amount of C‐C/C‐H groups of GO decreased and C—O—C/C—OH groups increased in used GO sample.

**Table 3:** Deconvoluted core level XPS spectral data of unused and used GO, Eu‐doped ZnO, and Eu‐doped ZnO@GO(used) at their BE of C1s, O1s, Zn2p, Eu3d, and Eu4d

	O 1s XPS spectra at BE [eV] (% composition)		C 1s XPS spectra at BE [eV] (% composition)		Zn 2p XPS spectra at BE [eV] (% composition) Spin‐orbital splitting energy [eV]	
Sample	Unused	Used	Unused	Used	Unused	Used
GO	O_OH_ 538.55 (35.09%)	O_OH_ 531.92 (28.77%)	C—C/C—H 284.2 (77.71%)	C—C/C—H 283.94 (42.57%)		
	O_v_ 532.81 (1145%)	O_v_ 532.56 (43.97%)	C—O—C 285.17 (7.69%)	C—O—C 284.63 (41.13%)		
	O_L 17.17%_	O_L_ 533.35 (27.25%)	C—O 286.07 (14.61%)	C—O 286.20 (16.29%)		
Eu‐doped ZnO	O_OH_ 528.78 (38.26%)	O_OH_ 531.08 (75.51%)	–	–	2p_3/2_ 1021.75 (65.74%)	2p_3/2_1021.10 (36.08%) 1024.40(20.24%)
	O_v_ 529.86 (15.41%)	O_v_ 532.82 (1.56%)			2p_1/2_ 1044.00 (34.58%)	2p_1/2_ 1044.30(26.94%) 1047.30 (16.74%)
	O_L_ 530.42 (48.32%)	O_L_ 534.13 (22.93%)			Δ = 23.2	Δ = 23.2; Δ = 22.9
Eu‐doped ZnO@GO	O_OH_ 532.09 (35.90%)	O_OH_ 530.70 (61.02%)	C—C/C—H 284.0 (72.88%)	C—C/C—H 283.78 (46.06%)	2p_3/2_ 1021.75 (70.32%)	2p_3/2_ 1021.70 (66.15%)
	O_v_ 531.54 (15.71%)	O_v_ 531.74 (21.31%)	C—O—C 284.98 (5.96%)	C—O—C 286.10 (49.84%)	2p_1/2_ 1044.90 (29.68%)	2p_1/2_ 1044.80 (33.85%)
	O_L_ 530.32 (48.40%)	O_L_ 531.88 (17.67%)	C—O 285.24 (23.58%)	C—O 288.28 (4.10%)	Δ = 23.15	Δ = 23.10

Short Eu^2+^ and Eu^3+^ peaks observed for the Eu‐doped ZnO(used) and the Eu‐doped ZnO@GO(used) in compared to their unused counterparts without major changes in their positions that revealed the some Eu^2+^ and Eu^3+^ species might be flused out in washing.

### 3.6 Surface Photovoltage Spectroscopy

**Figure 14:**
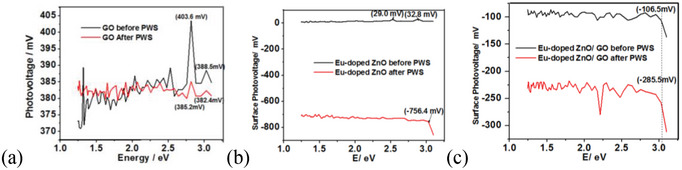
Surface photovoltage spectroscopic spectra for a) GO, b) Eu‐doped ZnO, and c) Eu‐doped ZnO@GO films, observed before and after PWS.

Surface photovoltage spectroscopic (SPS) spectra (**Figure** **14**a–c and **Table** **4**) before and after being utilized in PWS were used to evaluate the charge transfer process in the GO, Eu‐doped ZnO nanoassembly, and Eu‐doped ZnO/GO nanocomposite. These investigations may educate us about the photochemical charge transfer at solid–solid interfaces,^[^ ^73^ ^]^ low optical cross‐sectional states, trapping sites, and defect states due to the high sensitivity.^[^ ^74^ ^]^ Pure GO surfaces (black) and GO surfaces after being employed in PWS (red) both produce positive signals under visible light illumination with onsets at 1.302 eV and maximum photovoltages of Contact potential Difference (CPD) is 403.6 and 386.4 V at 2.818 eV, respectively. If the measured photovoltage is positive, then either electrons are being carried away from the substrate and moving in the direction of the Kelvin probe or the holes are being transported toward the substrate. That made GO's p‐type character apparent in both scenarios.^[^ ^75^ ^]^ While the Eu‐doped ZnO exhibits a downward slope and a negative photovoltage with a p‐type character before PWS and n‐type character after PWS. Additionally, the n‐type behavior with a sharp downward slope and negative photovoltage was evident in both in Eu‐doped ZnO and Eu‐doped ZnO@GO samples. That proved that GO supports the injection of specific holes into Eu‐ZnO to promote hole accumulation. This implies that a p–n‐heterojunction will form at the interface of Eu‐doped ZnO and GO in composite, by suppressing charge recombination. The optical bandgap (*E*_f_ = 2.21 eV Fermi level, 0.81 eV) and photoonset (3.02 eV) are in agreement that confirm the charge carriers are formed by bandgap excitation.

**Table 4:** Photovoltage observed for a) GO b) Eu‐doped ZnO and c) Eu‐doped ZnO@GO films, before and after PWS

Samples	Fresh samples used in PWS		Samples after used in PWS	
	Photovoltage (onset) at 3.02 eV mV^−1^	Photovoltage at 1.60 eV mV^−1^	Photovoltage (onset) at 3.02 eV mV^−1^	Photovoltage at 1.60 eV mV^−1^
GO	388.6	380.0	382.4	382.1
Eu‐doped ZnO	32.8	9.1	−756.4	−706.9
Eu‐doped ZnO@GO	−106.5	−95.0	−285.5	−233.0

GO, the electronically isolated components, supports to inject the selective holes from GO to Eu‐ZnO. This suggests the formation of a p–n‐heterojunction at the interface. The photopotential of this junction can be estimated as the difference between the contact potential of the Eu‐ZnO/GO sample and of the pure GO and Eu‐ZnO components.

**Figure 15:**
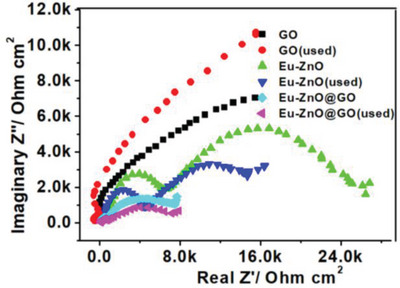
EIS Nyquist plots of the GO, Eu‐ZnO, and Eu‐doped ZnO@GO (before and after used in PWS applications).

EIS analysis was carried out to examine the charge seperation and transfer efficiency of the charge carriers. **Figure** **15** shows the two semicircles in the investigated‐frequency area of the Nyquist plots of the GO, Eu‐ZnO, and Eu‐doped ZnO@GO (fresh and used samples in PWS application). The electrode resistance, the charge transfer resistance for pseudocapacitive charge storage, including redox reactions and/or ion intercalation, or the electrolyte resistance in the porous electrodes have all been suggested as causes for the variation in diameter of the semicircles at higher frequencies shown in the Nyquist plots. Which demonstrates improved charge carrier separation and decreased interfacial charge transfer resistance.^[^ ^76^ ^]^ As a result, the EIS findings showed that Eu‐ZnO@GO underwent less charge‐recombination than GO and Eu‐ZnO, which enhanced the corresponding photocatalytic water splitting. Where the diameter of the s which demonstrates improved charge carrier separation and decreased interfacial charge transfer resistance.^[^ ^76^ ^]^ As a result, the EIS results showed that Eu‐ZnO@GO experienced less charge‐recombination than GO and Eu‐ZnO, which enhanced the associated photocatalytic water splitting. Where the diameter of the semicircle at lower frequencies represents the resistance of the solid electrolyte interphase layer, the ionic or diffusion resistance of the electrolyte, or the charge transfer resistance. Semicircle at lower frequencies is indicative of the ionic or diffusion resistance of the electrolyte, the resistance of the solid‐electrolyte interphase layer, or the charge transfer resistance.^[^ ^77^, ^78^ ^]^ After the semicircle plot, the graph turns upward at low frequencies, which is related to the ion transport limit of the electrolyte in porous electrode designs.^[^ ^79^ ^]^

The results showed that compared to their pure counterparts, the reused‐samples had increased interfacial charge transfer resistance. In contrast to the used samples, the fresh samples show better charge separation and transport. Comparing the observations of GO, Eu‐ZnO, and Eu‐doped ZnO@GO, it was found that the Eu‐doped ZnO@GO exhibits the lowest interfacial charge transfer resistance because it generates more photoelectrons than GO and Eu‐ZnO, which effectively separates and transports charges through the p–n junction. It shows the enhanced charge carrier segregation and diminished interfacial charge transfer resistance.^[^ ^76^ ^]^ Since then the Eu‐ZnO@GO had less charge‐recombination than the GO and Eu‐ZnO, the corresponding photocatalytic water splitting was improved, was in accordance to the EIS data (Table 4).

### 3.7 Hydrogen Generation through Water Splitting

The traditional approach was used to create the Eu‐doped ZnO loaded graphene oxide nanocomposite (Eu‐doped ZnO@GO), in order to enhance the hydrogen generating capabilities of the graphene oxide and Eu‐doped ZnO. Graphene oxide is employed to increase the surface area and serves as a bridge for electron transfers, while europium doping surpasses the visible light harvesting limit of the ZnO. All of these changes have been made to improve the charge separation effectiveness of molecular devices, which has improved the composite's ability to evolve hydrogen. The 0.30 g of as‐manufactured samples were employed to produce hydrogen through water splitting under 300 W Xe light source irradiation in 120 mL of 20% aqueous methanol.

**Figure 16:**
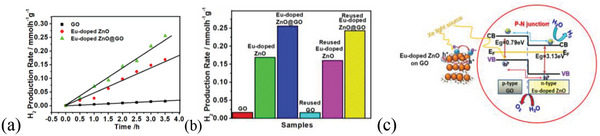
a) Hydrogen generation rate for GO, Eu‐doped ZnO, and Eu‐doped ZnO@GO at different time intervals and b) hydrogen generation rate for GO, Eu‐doped ZnO, and Eu‐doped ZnO@GO first time used and reused samples. c) Electron transfer mechanism for the molecular device Eu‐doped ZnO@GO.

In compared to the Eu‐doped ZnO's 168.7 µmol g^−1^ h^−1^ and GO's 16.07 µmol g^−1^ h^−1^, the nanocomposite Eu‐doped ZnO @GO had a good hydrogen generation capacity of 255.8 µmol g^−1^ h^−1^ (**Figure** **16**a,b). The below mentioned formula in Equation (6)^[^^8^ ^0^^]^ was used to compute the apparent quantum yield of hydrogen production rate of PWS, which is dependent on the measurement techniques, and conditions.

(6) $$\begin{array}{c} AQY=\frac{\left[number\;of\;reacted\;electrons\;or\;holes\right]}{\left[number\;of\;incident\;photons\right]\text{​}}\\ =\frac{[number\;of\;hydrogen\;molecules\;evolved\times 2]\times 100}{\left[number\;of\;incident\;photons\right]} \end{array}$$

where energy *E* is *nhν*, *n* is a number of incident photons, *h* is Planck constant, and ν is a frequency. For the used light source, the number of photons per second is 10^22^.

The composite shows the 6.47% apparent quantum efficiency for the system at 420 nm. The improved light harvesting capability, charge transfer capacity, and minimal flaws may be responsible for the rise in hydrogen generation with the advancement of the graphene oxide‐based devices. The suggested charge transfer mechanism for photocatalytic water splitting is mentioned by Equations (7)–(10).

(7) $$\begin{array}{c} Eu^{3+}-doped\;ZnO@GO\to (e^{-}+h^{+}){\text{Eu}}^{3+}-doped\;ZnO@GO \end{array}$$

(8) $$\begin{array}{c} Eu^{3+}-doped\;ZnO@GO+e^{-}\to Eu^{2+}-doped\;ZnO@GO \end{array}$$

(9) $$\begin{array}{c} Eu^{2+}-doped\;ZnO@GO\to Eu^{3+}-doped\;ZnO@GO\left(e^{-}\right) \end{array}$$

(10) $$\begin{array}{c} \begin{array}{l} 2{\text{Eu}}^{3+}-doped\;ZnO@GO\left(e^{-}\right)+2H^{+}\to \\ \;2{\text{Eu}}^{3+}-doped\;ZnO@GO+H_{2} \end{array} \end{array}$$

(11) $$\begin{array}{c} \begin{array}{l} Eu^{3+}-doped\;ZnO@GO\left(h^{+}\right)+{\text{CH}}_{3}OH\to \\ \;Eu^{2+}-doped\;ZnO@GO+HCHO+2H^{+} \end{array} \end{array}$$

(12) $$\begin{array}{c} \begin{array}{l} Eu^{3+}-doped\;ZnO@GO\left(h^{+}\right)+{\text{CH}}_{3}OH\to \\ \;Eu^{2+}-doped\;ZnO@GO+CO+2H^{+} \end{array} \end{array}$$

(13) $$\begin{array}{c} \begin{array}{l} Eu^{2+}-doped\;ZnO@GO+{\text{CH}}_{3}OH+CO\to \\ \;Eu^{2+}-doped\;ZnO@GO+{\text{CH}}_{3}COOH \end{array} \end{array}$$

(14) $$\begin{array}{c} \begin{array}{l} Eu^{2+}-doped\;ZnO@GO\left(h^{+}\right)+{\text{CH}}_{3}COOH+H_{2}O\to \\ \;Eu^{2+}-doped\;ZnO@GO+HCOOH+{\text{CH}}_{3}OH \end{array} \end{array}$$

The electron transport mechanism of the molecular device, which can generate a p–n junction at the interface of the Eu‐doped ZnO and GO, is shown in Figure 16c. In this case, GO serves as a p‐type material and Eu‐doped ZnO serves as an n‐type material. The electron tends to go from valance band (VB) to CB when exposed to light. In order to convert water into hydrogen gas, the as‐approached CB electrons at the GO site were commanded to be transported toward the CB of Eu‐doped ZnO. Additionally, the CH_3_OH was oxidized into various products at the holes left at the VB sites of the Eu‐doped ZnO and GO (HCOOH, HCHO, CO_2_, etc.).

## 4 Conclusions

In conclusion, the original Eu‐doped ZnO assembly was modified by the traditional hydrothermal process that used to create the Eu‐doped ZnO@GO nanocomposite, (Eu‐doped ZnO tethered to GO by ultrasonication method). Additionally, the composite Eu‐doped ZnO@GO results in a greater charge separation, a decrease in Eu^3+^ concentration, and a smaller defect density. In this case, graphene oxide can offer a significant surface area with active sites and function as a good electron transfer mediator as well as it sustains the stability of the nanoassembly Eu‐doped ZnO otherwise ZnO in assembly, gone through the break down in to Zn^2+^ and Zn^0^(XPS supported) after PWS. Europium acts as a photosensitizer in Eu‐doped ZnO. All of these advancements used to increase the composite's capability of hydrogen evolution and charge separation. The interface in Eu‐doped ZnO@GO nanocomposites work as n‐p‐type semiconductor where Eu‐doped ZnO act as a n‐type semiconductor and GO as a p‐type semiconductor, respectively. The heterojunction in nanocomposite functions as a p–n junction to facilitate the charge separation. Finally, as‐synthesized nanocomposite was tested for hydrogen evolution through photocatalytic water splitting under 300 W Xe light exposure and produce 1.52 times higher hydrogen gas than the Eu‐doped ZnO in 20% aqueous methanol.

## 5 Experimental Section

### Synthesis of Graphene Oxide

GO was fabricated by adapting Hummer's modified approach. In which 36NH_2_SO_4_ (46 mL) in a 500 mL beaker was added to the graphite powder (2 g) and NaNO_3_ (1 g), which were then agitated for 15 min in an ice bath. Next, KMnO_4_ (6 g) was slowly added to the aforementioned mixture. The resulting solution was swirled and thoroughly mixed with distilled water (92 mL) for 2 h at room temperature, then allowed to settle for 30 min. After the addition of distilling water (280 mL) and 20 mL of 30% H_2_O_2_, the mixture was stirred for further 1 h. The suspension was then repeatedly rinsed with water until the solution's pH reached 7. The solution was gently sonicated for 30 min at room temperature to exfoliate the GO as dark brown powder, which was dried in an air‐oven at 60 °C for overnight.

### Synthesis Eu‐Doped Zno Nanoassembly

The Eu‐doped ZnO nanoassembly was prepared using the hydrothermal technique. In which liquid ammonia (NH_4_OH) was added to 40 mL of aqueous Zn(NO_3_)_2_.6H_2_O (2.67 g) and Eu(NO_3_)_2_.6H_2_O (0.40 g) until the pH of the solution reached 7. The produced slurry of Eu‐doped ZnO was transferred to a 100 mL Teflon‐lined stainless‐steel autoclave, which contained 35 mL of 20% CTAB. The autoclave was carefully sealed and it was then heated at 200 °C for 10 h in an air‐oven. The autoclave was then allowed to gradually drop to room temperature, and the final solution was filtered before being repeatedly washed in both water and alcohol before being dried at 60 °C for 24 h.

### Synthesis of Eu‐Doped ZnO/GO Nanocomposite

As it was being produced, the 0.5 g of Eu‐doped ZnO nanoassembly was introduced in 50 mL of water, which was then sonicated for 15 min. In a separate beaker, 0.5 g of GO suspension in Deionised water (DIW) was sonicated for 15 min. Afterward both of the two suspensions are then combined and sonicated for 1 h, then agitated for 2 h. The final product is filtered, and washed numerous times with DIW and ethanol, and dried at 60 °C for 24 h. The water splitting properties of the as‐synthesized GO, Eu‐doped ZnO nanoassembly, and Eu‐doped ZnO@GO nanocomposites were investigated.

### Electrode Preparation

Working electrodes were created using the electrode materials, i.e., GO, Eu‐doped ZnO nanoassembly, and Eu‐doped ZnO@GO nanocomposite for electrochemical study. The 0.30 g of the as‐prepared material was grinded with 5 mL of acetyl acetone: water (1.5:8.5 v/v) solution containing 0.2 g of Triton‐100 as binder. The 1 × 1 cm^2^ area of Florine‐doped Tin oxide (FTO) glass was casted with the as‐prepared mixture, which was connected to copper wire through the silver paste, and the remaining area was covered with epoxy resin. The electrode was dried overnight at 60 °C in a vacuum oven. Then as fabricated electrodes were heated at 450 °C at 0.5 h.

## Conflict of Interest

The authors declare no conflict of interest.

## Author Contributions

The author contribution is as follows: investigations and writing (N.G.) and supervision, interpretation of results, and conceptualization (N.C.).

## Supporting information

Supporting Information

## Data Availability

The data that support the findings of this study are available from the corresponding author upon reasonable request.

## References

[1] A. K. Geim , K. S. Novoselov , in Nanoscience and Technology: A Collection of Reviews From Nature Journals (Ed: P. Rodgers ), World Scientific, Singapore 2010, pp. 11–19.

[2] J. Luo , J. Kim , J. Huang , Acc. Chem. Res. 2013, 46, 2225. 23425088 10.1021/ar300180n

[3] T. S. Sree‐prasad , V. Berry , Small 2013, 9, 341.23169614

[4] Y. Xu , H. Bai , G. Lu , C. Li , G. Shi , J. Am. Chem. Soc. 2008, 130, 5856. 18399634 10.1021/ja800745y

[5] X. Huang , Z. Yin , S. Wu , X. Qi , Q. He , Q. Zhang , H. Zhang , Small 2011, 7, 1876. 21630440 10.1002/smll.201002009

[6] D. Chen , H. Feng , J. Li , Chem. Rev. 2012, 112, 6027. 22889102 10.1021/cr300115g

[7] D. R. Dreyer , S. Park , C. W. Bielawski , R. S. Ruoff , Chem. Soc. Rev. 2010, 39, 228. 20023850 10.1039/b917103g

[8] C. Gómez‐Navarro , R. T. Weitz , A. M. Bittner , M. Scolari , A. Mews , M. Burghard , K. Kern , Nano Lett. 2007, 7, 3499. 17944526 10.1021/nl072090c

[9] C. Gómez‐Navarro , J. C. Meyer , R. S. Sundaram , A. Chuvilin , S. Kurasch , M. Burghard , U. Kaiser , Nano Lett. 2010, 10, 1144. 20199057 10.1021/nl9031617

[10] F. Tuinstra , J. L. Koenig , J. Chem. Phys. 1970, 53, 1126.

[11] T. F. Yeh , F. F. Chan , C. T. Hsieh , H. Teng , J. Phys. Chem. C 2011, 115, 22587.

[12] D. S. Sutar , G. Singh , V. Divakar Botcha , Jpn. J. Appl. Phys., Part 2 2012, 101, 103103.

[13] I. V. Lightcap , T. H. Kosel , P. V. Kamat , Nano Lett. 2010, 10, 577. 20055433 10.1021/nl9035109

[14] Y. H. Ng , A. Iwase , A. Kudo , R. Amal , Solid State Commun. 2010, 1, 2607.

[15] G. Williams , B. Seger , P. V. Kamat , ACS Nano 2008, 2, 1487. 19206319 10.1021/nn800251f

[16] H. Zhang , X. Lv , Y. Li , Y. Wang , J. Li , ACS Nano 2010, 4, 380. 20041631 10.1021/nn901221k

[17] W. Zhou , T. Li , J. Wang , Y. Qu , K. Pan , Y. Xie , H. Fu , Nano Res. 2014, 7, 731.

[18] P. Li , H. Liu , F. X. Xu , Y. Wei , Mater. Chem. Phys. 2008, 112, 393.

[19] W. Cun , Z. Jincai , W. Xinming , M. Bixian , S. Guoying , P. Ping'an , F. Jiamo , Appl. Catal., B 2002, 39, 269.

[20] S. Liu , C. Li , J. Yu , Q. Xiang , CrystEngComm 2011, 13, 2533.

[21] 21. W. J. Sun , J. Li , G. Mele , Z. Q. Zhang , F. X. Zhang , J. Mol. Catal. A: Chem. 2013, 366, 84.

[22] Y. Min , K. Zhang , Y. Chen , Y. Zhang , W. Zhao , Sep. Purif. Technol. 2012, 92, 115.

[23] P. Malathy , K. Vignesh , M. Rajarajan , A. Suganthi , Ceram. Int. 2014, 40, 101.

[24] N. R. Khalid , E. Ahmed , Z. Hong , M. Ahmad , Appl. Surf. Sci. 2012, 263, 254.

[25] I. Ahmad , M. S. Akhtar , E. Ahmed , M. Ahmad , V. Keller , W. Q. Khan , N. R. Khalid , Sep. Purif. Technol. 2020, 237, 116328.

[26] K. T. Ranjit , I. Willner , S. H. Bossmann , A. M. Braun , Environ. Sci. Technol. 2001, 35, 1544. 11348099 10.1021/es001613e

[27] R. Sasikala , V. Sudarsan , C. Sudakar , R. Naik , T. Sakuntala , S. R. Bharadwaj , Int. J. Hydrogen Energy 2008, 33, 4966.

[28] I. Ahmad , S. Shukrullah , M. Y. Naz , E. Ahmed , M. Ahmad , Int. J. Hydrogen Energy 2022, 47, 15505.

[29] H. Cho , H. Joo , H. Kim , J. E. Kim , K. S. Kang , J. Yoon , Appl. Surf. Sci. 2021, 565, 150459.

[30] F. J. Sheu , C. P. Cho , Y. T. Liao , C. T. Yu , Catalysts 2018, 8, 57.

[31] C. Cui , S. Li , Y. Qiu , H. Hu , X. Li , C. Li , W. Tang , Appl. Catal., B 2017, 200, 666.

[32] M. Cao , P. Wang , Y. Ao , C. Wang , J. Hou , J. Qian , Int. J. Hydrogen Energy 2015, 40, 1016.

[33] D. Geetha , T. Thilagavathi , Dig. J. Nanomater. Biostruct. 2010, 5, 297.

[34] D. Li , M. B. Müller , S. Gilje , R. B. Kaner , G. G. Wallace , Nat. Nanotechnol. 2008, 3, 101. 18654470 10.1038/nnano.2007.451

[35] H. J. Zhai , W. H. Wu , F. Lu , H. S. Wang , C. Wang , Mater. Chem. Phys. 2008, 112, 1024.

[36] D. Yiamsawas , K. Boonpavanitchakul , W. Kangwansupamonkon , J. Microsc. Soc. Thailand 2009, 23, 75.

[37] S. Y. Chu , T. M. Yan , S. L. Chen , J. Mater. Sci. Lett. 2000, 19, 349.

[38] H. Kumar , R. Rani , Int. Lett. Chem., Phys. Astron. 2013, 14, 26.

[39] H. Hønsi , C. Kjetland , S. Liautaud , Rendez‐vous Oslo Cappelen1, 2006

[40] A. Sawby , M. Selim , S. Marzouk , M. Mostafa , A. Hosny , Physica B: Phys. Condens. Matter 2010, 405, 3412.

[41] D. Zhang , Z. Xue , Q. Wang , J. Phys. D 2002, 35, 2837.

[42] G. Eda , Y. Y. Lin , C. Mattevi , H. Yamaguchi , H. A. Chen , I. S. Chen , M. Chhowalla , Adv. Mater. 2010, 22, 505. 20217743 10.1002/adma.200901996

[43] M. Mermoux , Y. Chabre , A. Rousseau , Carbon 1991, 29, 469.

[44] W. Cai , R. D. Piner , F. J. Stadermann , S. Park , M. A. Shaibat , Y. Ishii , R. S. Ruoff , Science 2008, 321, 1815. 18818353 10.1126/science.1162369

[45] L. Verbit , J. Am. Chem. Soc. 1965, 87, 1617.

[46] H. Hosoya , J. Tanaka , S. &Nagakura , J. Mol. Spectrosc. 1962, 8, 257.

[47] H. A. Becerril , J. Mao , Z. Liu , R. M. Stoltenberg , Z. Bao , Y. Chen , ACS Nano 2008, 2, 463. 19206571 10.1021/nn700375n

[48] S. Dubin , S. Gilje , K. Wang , V. C. Tung , K. Cha , A. S. Hall , R. B. Kaner , ACS Nano 2010, 4, 3845. 20586422 10.1021/nn100511a PMC3939021

[49] S. Stankovich , D. A. Dikin , R. D. Piner , K. A. Kohlhaas , A. Kleinhammes , Y. Jia , R. S. Ruoff , Carbon 2007, 45, 1558.

[50] D. Yang , A. Velamakanni , G. Bozoklu , S. Park , M. Stoller , R. D. Piner , R. S. Ruoff , Carbon 2009, 47, 145.

[51] D. Li , R. B. Kaner , Science 2008, 320, 1170. 18511678 10.1126/science.1158180

[52] O. Akhavan , E. Ghaderi , J. Phys. Chem. C 2009, 113, 20214.

[53] J. Wang , L. Gao , Solid State Commun. 2004, 132, 269.

[54] X. L. Wu , G. G. Siu , C. L. Fu , H. C. Ong , Appl. Phys. Lett. 2001, 78, 2285.

[55] W. Chen , J. O. Malm , V. Zwiller , Y. Huang , S. Liu , R. Wallenberg , L. Samuelson , Phys. Rev. B 2000, 61, 11021.

[56] C. A. Nascimento , A. B. Schura , E. F. Chagas , R. J. Ramos , H. de Santana , A. Marletta , E. M. Therézio , J. Mater. Sci.: Mater. Electron. 2020, 31, 6629.

[57] Phosphor Handbook (Eds: S. Shionoya , W. M. Yen ), CRC Press, Boca Raton, FL 1999.

[58] G. Blasse , B. C. Grabmaier , in Luminescent Materials, Springer, Berlin 1994, pp. 1–9.

[59] V. Pike , S. Patraw , A. L. Diaz , B. G. de Boer , J. Solid State Chem. 2003, 173, 359.

[60] M. Nogami , T. Yamazaki , Y. Abe , J. Lumin. 1998, 78, 63.

[61] P. Boolchand , K. C. Mishra , M. Raukas , A. Ellens , P. C. Schmidt , Phys. Rev. B 2002, 66, 1344.

[62] G. C. Smith , J. Electron Spectrosc. Relat. Phenom. 2005, 148, 21.

[63] R. Vercaemst , D. Poelman , L. Fiermans , R. L. Van Meirhaeghe , W. H. Laflere , F. Cardon , J. Electron Spectrosc. Relat. Phenom. 1995, 74, 45.

[64] M. Chen , X. Wang , Y. H. Yu , Z. L. Pei , X. D. Bai , C. Sun , L. S. Wen , Appl. Surf. Sci. 2000, 158, 134.

[65] S. Major , S. Kumar , M. Bhatnagar , K. L. Chopra , Jpn. J. Appl. Phys., Part 2 1986, 49, 394.

[66] P. D. Maker , R. W. Terhune , M. Nisenoff , C. M. Savage , Phys. Rev. 1962, 8, 21.

[67] C. C. Zheng , S. J. Xu , J. Q. Ning , S. F. Zhang , J. Y. Wang , C. M. Che , J. H. Hao , J. Appl. Phys. 2011, 109, 013528.

[68] W. N. Herman , L. M. Hayden , J. Opt. Soc. Am. B 1995, 12, 416.

[69] P. D. B. Vincent Crist , Handbook of Monochromatic XPS Spectra—The Elements and Native Oxides, Vol. 1, XPS International, Inc, California 1999, p. 2.

[70] M. N. Islam , T. B. Ghosh , K. L. Chopra , H. N. Acharya , Thin Solid Films 1996, 280, 20.

[71] J. F. Moulder , W. F. Stickle , P. E. Sobol , K. D. Bomben , in Handbook of X‐Ray Photoelectron Spectroscopy (Eds: J. Chastain , R. C. King, Jr. ), Perkin‐Elmer Corporation, Physical Electronics, Inc, MinnCOIa, USA 1992.

[72] L. Dai , Acc. Chem. Res. 2013, 46, 31. 23030244 10.1021/ar300122m

[73] F. E. Osterloh , M. A. Holmes , L. Chang , A. J. Moule , J. Zhao , J. Phys. Chem. C 2013, 117, 26905.

[74] M. Waller , T. K. Townsend , J. Zhao , E. M. Sabio , R. L. Chamousis , N. D. Browning , F. E. Osterloh , Chem. Mater. 2012, 24, 698.

[75] a) R. Beranek , B. Neumann , S. Sakthivel , M. Janczarek , T. Dittrich , H. Tributsch , H. Kisch , Chem. Phys. 2007, 339, 11;

[76] M. Z. Bazant , K. Thornton , A. Ajdari , Phys. Rev. E: Stat., Nonlinear, Soft Matter Phys. 2004, 70, 021506. 10.1103/PhysRevE.70.021506 15447495

[77] S. Wang , B. Hsia , C. Carraro , R. Maboudian , J. Mater. Chem. A 2014, 2, 7997.

[78] R. Thangappan , S. Kalaiselvam , A. Elayaperumal , R. Jayavel , M. Arivanandhan , R. Karthikeyan , Y. Hayakawa , J. Phys. Chem. C 2018, 122, 24499. 10.1039/c5dt04832j 26732466

[79] Y. Huang , Y. Li , Z. Hu , G. Wei , J. Guo , J. Liu , J. Mater. Chem. A 2013, 1, 9809.

[80] A. Kudo , H. Kato , S. Nakagawa , J. Phys. Chem. B 2000, 104, 571.

