# Supplementary material for: P–N Heterojunction System Eu‐Doped ZnO@GO for Photocatalytic Water Splitting
Source: Glob Chall. 2023 Jan 22;7(4):2200106. doi: 10.1002/gch2.202200106 (PMC10069305; doi:10.1002/gch2.202200106)
Supplement: Supplementary file 1 — Supporting Information [file GCH2-7-2200106-s001.pdf]

Global Challenges
Open Access

Supporting Information

for *Global Challenges,* DOI: 10.1002/gch2.202200106

P–N Heterojunction System Eu-Doped ZnO@GO for Photocatalytic Water Splitting

Neeta Gurbani and Neelu Chouhan**

Supplementary Information

p-n Heterojunction system Eu-doped ZnO@GO for photocatalytic water splitting

Neeta Gurbani,[a] and Neelu Chouhan[a*]

[a]*Department of Pure and Applied Chemistry, University of Kota, MBS Road, Kota-324005 (India) email: chgurbani@gmail.com and neeluchouhan@uok.ac.in*

XPS of GO (unused)

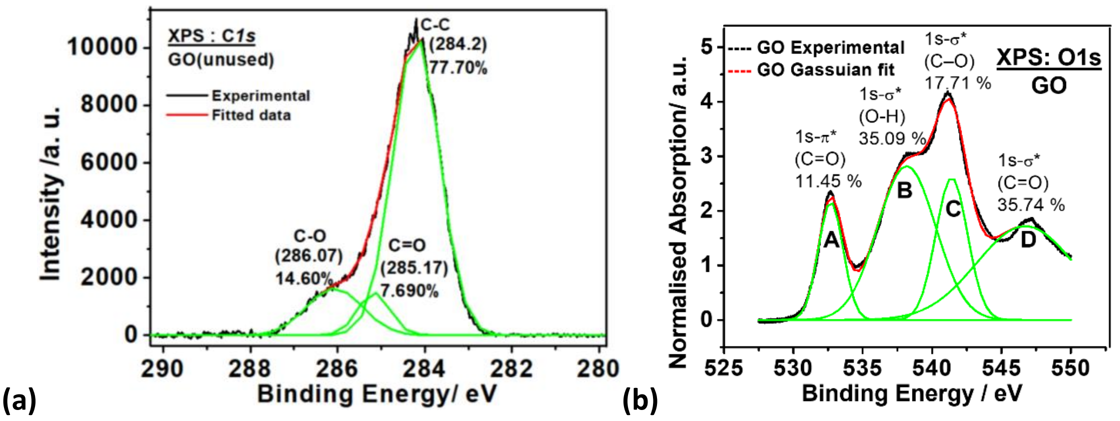

Figure S1. Deconvoluted core level XPS spectra of GO at binding energy of (a) C*1s*
